# Use of health services and medication use, new comorbidities, and mortality in patients with chronic diseases who did not contract COVID-19 during the first year of the pandemic: a retrospective study and comparison by sex

**DOI:** 10.1186/s12913-023-10158-7

**Published:** 2023-12-06

**Authors:** Liliana Mahuela, Bárbara Oliván-Blázquez, Ana Lear-Claveras, Fátima Méndez-López, Mario Samper-Pardo, Sandra León-Herrera, Rosa Magallón-Botaya, María Antonia Sánchez-Calavera

**Affiliations:** 1 Institute for Health Research Aragon (IISAragon), Zaragoza, Spain; 2 Aragones Group of Research in Primary Health Care (GAIAP), Zaragoza, Spain; 3 Department of Psychology and Sociology, University of Zaragoza, Zaragoza, Spain; 4 Department of Medicine, University of Zaragoza, Zaragoza, Spain

**Keywords:** Chronic Diseases, Comorbidity, Gender, Lockdown, COVID-19 pandemic, Health services, Primary health care

## Abstract

**Background:**

The restrictions introduced to stop the spread of the COVID-19 virus have also had a direct impact on people with chronic diseases and especially on diseases to which lifestyles are relevant in their control and management, such as diabetes, hypertension, chronic obstructive pulmonary disease (COPD), etc. The purpose of this paper is to conduct a longitudinal analysis of new comorbidities, mortality, medication use, and the use of health resources in patients with chronic diseases who did not contract COVID-19, comparing the six months before the strict lockdown to the 12 months following the end of the strict lockdown.

**Method:**

An observational real world data pre-post study of 668,974 people was undertaken. The patients studied were over 16 years of age, had been receiving care from the Aragon Health Service (Northeastern Spain), were diagnosed with one or more chronic diseases, and had not contracted COVID-19. Sociodemographic, comorbidity, pharmacological and health resource use variables were collected during the six months before the onset of the lockdown and during the six and 12 months following the end of the lockdown. The comparisons by sex were carried out using a Student T-test or chi-squared test to analyse differences.

**Results:**

Dyslipidaemia (42.1%) followed by hypertension (35.1%) and anxiety and depression (34.6%) were the most prevalent chronic diseases among the study population. 78.% of patients had between one and four chronic illnesses. There was a decrease in new diagnoses of other chronic comorbidities in this population and a decrease in medications prescribed and the use of health services.  Although women received more diagnoses of chronic diseases, the number of medications dispensed was lower, but the use of health services was higher. These figures were maintained throughout the pandemic.

**Conclusion:**

Our results suggest an underdiagnosis of new chronic comorbidities and a decrease in mortality rates from causes unrelated to COVID-19 due to the closure of health centres in Aragón (Spain) during the lockdown. This trend was exacerbated in women. The underdiagnosis of new chronic comorbidities during confinement can cause the disease to worsen, with the consequent increase in symptoms and the worsening of chronic pathologies in patients with a severe evolution.

**Supplementary Information:**

The online version contains supplementary material available at 10.1186/s12913-023-10158-7.

## Background

In Spain, the population over the age of 74 is set to double in the next 40 years, and the dependency rate will rise to 89.6% from the current 47.8% [1]. This ageing population is associated with an increase in the number of people with chronic diseases (osteoarticular, cardiovascular, respiratory, mental, neurodegenerative, and cancer), with a consequent rise in multimorbidity (two or more concurrent conditions), mortality in the world [2]. In Spain, diseases of the circulatory system remained the leading cause of death in 2021, with 26.4% of the total (and a rate of 251.8 deaths per 100,000 inhabitants), followed by tumours, with 25, 2% of the total (and a rate of 240.1) [3]. They also significantly impact the daily lives of patients and their families, and their management is a challenge for society [4–6]. Patients with a higher number of associated comorbidities have increased health care and care needs compared to those who do not suffer from chronic conditions, which is confirmed through their high use of health services, especially in primary health care (PHC) [7].

When assessing the significant differences between the sexes, it is necessary to consider the conditioning of being a woman and their role in the family, social functioning, and job expectations. These simultaneous processes worsen and limit the health status of women alongside the influence of biological, psychological, and social factors that condition them [8, 9]. According to the Spanish National Institute of Statistics, 68.2% of women over 15 years have a chronic disease compared to 60% of men. This difference between the sexes is exacerbated in those with lower incomes, with 77.5% of women who had an income less than 1,050 euros presenting at least one chronic disease compared to 65% of men with the same level of income, coinciding with what was obtained in our results [10].

Around the world, the current COVID-19 pandemic has tested people, governments, and health systems’ ability to respond and adapt [11]. Its impact on essential health services has been notable worldwide. These services were modified as they were focused on detecting mild cases of the infection, following up on positive cases and contact tracing. The World Health Organization (WHO) conducted a PULSE survey assessing the interruptions to healthcare services. This survey showed that most countries (90%) have seen disruption to their essential healthcare services since the beginning of the pandemic [12]. The saturation of PHC services has interrupted the care of patients with chronic diseases.

Furthermore, to reduce the risk of COVID-19 transmission, medical appointments were postponed or carried out via telephone [13]. This reorientation of health systems reduced their ability to prevent or control chronic diseases that need regular assistance and care, especially in PHC [12, 14]. Several studies have been conducted that have confirmed the reduction of new diagnoses of the most prevalent pathologies, observing a decrease in the annual incidence rate for all processes studied in 2020 except for anxiety disorders. In diseases such as hypertension and COPD, the diagnosis rate decreased by 50% compared to the previous year. In the case of diabetes mellitus, heart failure, cancer, and strokes, it fell by approximately 10–15% [15].

The Spanish health system is a mixed system where the National Health System, managed by public entities, coexists with an extensive private hospital network. This high-quality health system guarantees almost universal coverage for all residents [16].

Over 70% of the healthcare system is financed by public taxes, which is reported to be around 10.7% of the gross domestic product [17]. It is common for some Spanish residents to have private health insurance to supplement public health coverage. In addition, Spain operates with a co-payment system for prescription medications. The contribution of citizens to pharmaceutical benefits is proportional to their level of income [16].

PHC services are the closest to the population and comprise the main form of healthcare in which most chronic diseases are managed.

Most studies on chronic diseases and COVID-19 have investigated the influence of COVID-19 on the prognosis of infected patients [18–21]. However, few have analysed the impact of the pandemic on patients with chronic conditions who have not been infected. It is necessary to carry out large-scale studies that provide a longitudinal perspective, considering the effects of confinement and the consequent health, social, political, and economic crises. An example is the retrospective observational study of population data linkage in Wales between 2000 and 2021 [22]. In addition, recent studies mostly have cross-sectional designs and small sample sizes and were predominantly conducted to analyse data during the first wave of COVID-19.

Therefore, this paper aims to study and conduct a longitudinal analysis of new comorbidities, mortality, the medication for chronic pathologies use and the use of health and resources of patients with chronic diseases in an autonomous community in Southern Europe (Aragon, Spain) who did not contract COVID-19, comparing by sex and three-time frames (the six months before the start of lockdown, six months after the end of the lockdown, and from six to twelve months following the end of the strict lockdown.

## Methods

### Design and study population

This research project was a retrospective observational study using real-world data (RWD) from three time frames (six months pre-pandemic, six months after the start of the strict lockdown and from six to 12 months following the end of the strict lockdown). There are 1,122,151 people over 16 years old registered in the Autonomous Region of Aragon, in Northeastern Spain, with medical records in PHC centres.

In Spain, 16 years is the minimum age for patients to be treated by a primary health care physician instead of a paediatrician for their usual treatment. This study’s final sample was made up of individuals aged over 16 years old with an electronic health record in the Aragon Health Service, a diagnosis of a chronic illness with a prevalence higher than 5% [23], according to the International Classification of Primary Care (ICPC-2) [24] who did not contract COVID-19 evaluated through the data of the electronic health record) (n = 668,974 subjects). The existence of positive COVID-19 diagnostic tests (antigen, polymerase chain reaction or serology) was evaluated in the electronic health record. Given that the healthcare system in Spain is universal, with practically no other primary healthcare providers, it is considered that the data obtained in this study are representative of almost 85% of th population who met the study’s inclusion criteria [25].

**Fig. 1:**
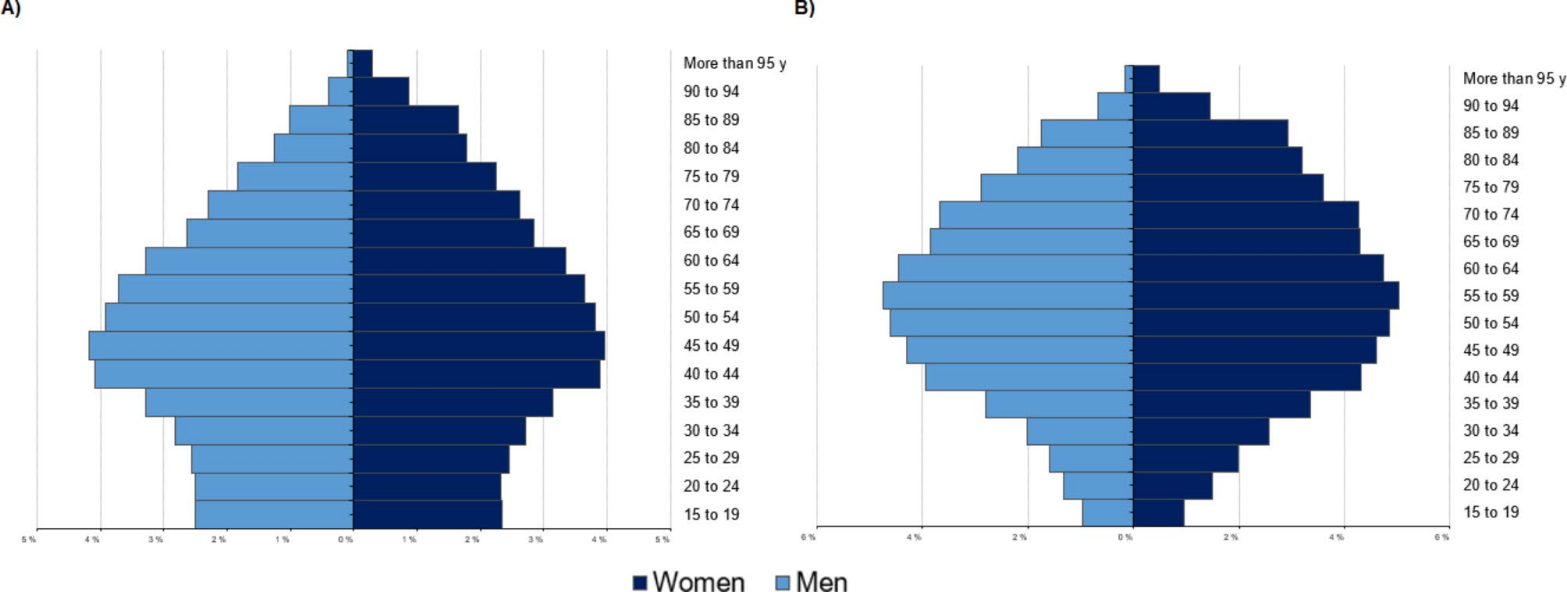
Aragonese Population Pyramids September 2019 Note **A)** Total Aragonese population September 2019 [26]; **B)** Population of sample study at baseline.

On March 15 2020, the Spanish Government declared a national emergency, limiting mobility and requiring the population to stay home until May 3. It was a strict lockdown and marked the beginning of the pandemic. Data were collected from each individual at different periods, including people who had not been infected with COVID-19 during this period or previously. The baseline measurement was taken six months before the lockdown (from September 14, 2019, to March 15 2020)(n = 662,754). The second measurement was taken in the six months following the end of the strict lockdown (from May 3 to November 4 2020) (n = 654,954), and the third measurement was taken from six to 12 months after the lockdown (from November 5 2020 to May 6 2021) (n = 649,702). Figure 1A shows the Aragonese population as of September 14, 2019 [26], and Fig. 1B shows the Aragonese population with some chronic pathology. In supplementary 1 are graphs of the study population that has not contracted COVID-19 versus those of the same age range that have suffered it in the three assessments.

### Variables

The variable data were collected from longitudinal electronic health records from Aragon’s PHC centres. The sociodemographic variables included in this study were sex, age, and pharmaceutical delivery, which is related to the patient’s income (< 18,000 euros/year; between 18,000 and 100,000 euros/year; >100,000 euros/year); other (free prescriptions, mutualist and uninsured) and residence defined as semiurban/rural or urban health area (less or more than 10,000 inhabitants, respectively). The number of deaths in the study population was also considered for each measurement period. Comorbidity with other chronic diseases was also considered during each period. Chronic conditions with a prevalence greater than 5% [23] were also included. The chronic diseases considered included arrhythmias, heart failure, ischaemic heart disease, hypertension, dyslipidaemia, obesity, vascular disease, cerebrovascular disease, diabetes, chronic bronchitis, chronic obstructive pulmonary disease, asthma, chronic kidney disease, smoking, alcoholism, insomnia, anxiety and depression, autolytic attempt, anaemia, neoplasia, dementia, hearing loss, cataracts, and glaucoma. New diagnoses of these comorbid conditions were also collected. Patient medication use over the different periods was collected, considering the number of active ingredients dispensed by the pharmacy and their cost. It was decided to consider the dispensed medication and not the prescribed one because some prescribed medications may not have been dispensed as the patient may not have picked them up from the pharmacy. Finally, the patient’s use of health and resources during the studied time frames was assessed using variables related to the use of PHC health and services (number of nurse and general practitioner (GP) visits for ordinary or continuous care at health centres or home and the number of visits to other health centre professionals such as physiotherapists, midwives, odontostomatologists and social workers) and the use of specialised hospital services (number of visits to specialised care units at hospital number of visits to the accident and emergency department (A&E), hospitalisations and admissions to the intensive care unit (ICU) as well as the duration of these stays).

### Statistical analysis

Given the large sample size, parametric statistics were used [27]. To determine the characteristics of the population in the analysed variables, a descriptive analysis of the study variables was carried out using frequencies (percentages) to summarise the categorical variables and measures of central tendency and dispersion (mean and standard deviation) for the continuous variables.

For the study population, mortality due to causes other than COVID-19 (as the patients did not contract COVID-19) was assessed by calculating the crude mortality rate for each of the three periods. The population of Aragon in the middle of each of the three periods was used as the denominator.

Differences in medication consumption were assessed (number of active ingredients and cost) in the study population during each period using a paired samples t-test. The mean and standard deviation of each variable of health and resources (primary and specialised hospital care) were also calculated. A paired samples t-test was used to compare the difference in the mean, too. Cochran’s Q was used to evaluate the differences in the % of new diagnoses between the three times. When the sample size was lower than 100 observations, the Wilcoxon test was used. This statistical test was used for analysing ICU variables since the number of observations was 15 patients (pre-six months) and 24 patients (six to twelve months). The comparisons by sex were calculated using a Student T-test or chi-squared test depending on the variable (continuous or categorical). All data were stored and analysed with databases using IBM SPSS Statistics software (version 25.0) [28].

### Ethical considerations

The Aragón Clinical Research Ethics Committee reviewed and approved the studies involving human participants. (Protocol code PI20/175, approval date May 13 2020). All procedures contributing to this work comply with the ethical standards of the Aragon Clinical Research Ethics Committee (part of the Government of Aragon’s Department of Health) and with the 1975 Helsinki Declaration, revised in 2008. Data were obtained from clinical records provided in a non-identifiable format by the Aragonese Health Service. Written informed consent from the participants or their legal guardian/next of kin was not required to participate in this study by the national legislation and the institutional requirements (Law 14/2007, of July 3, on Spanish Biomedical Research). The European Parliament’s 2016/679 Regulation (EU) and the 3/2018 Spanish Organic Law on the Protection of Personal Data and the Guarantee of Digital Rights processed, notified and transferred personal data.

## Results

On September 14 2019, 732,585 people in Aragon were over 16 years old and diagnosed with a chronic pathology in their electronic health record. Of these people, 668,974 did not contract COVID-19 during the first year of the pandemic (as of May 6, 2021), which are included in the study. Of these patients, 307,823 (46%) were men and 361,151 (54%) were women, and the mean age of the sample was 56.74 years (SD: 18.48) with an age range from 16 to 108 years old. Two-thirds (69.5%) had an annual income below 18,000 euros, and more than half (53.2%) resided in urban areas (with more than 10,000 inhabitants) [Table 1]. Dyslipidaemia (42.1%), followed by hypertension (35.1%) and anxiety and depression (34.6%), were the most prevalent chronic diseases among the patients in the study. 75.2% of patients had between 1 and 4 chronic illnesses. Depression, anxiety, and anaemia are more frequent in women, whereas ischaemic heart disease, dyslipidaemia, COPD, smoking, and alcoholism are more frequent in men. 16% of women have one unique chronic disease versus 25% of men. However, 54% of women and men have between 2 and 4 chronic diagnoses [Table 1].

**Table 1:** Pre-lockdown sociodemographic data and chronic illness in chronic patients uninfected with COVID-19.

	Total n = 668,974	Women n = 361,151	Men n = 307,823	P-value
**Age**, Mean ± SD	56.74 ± 18.48	57.51 ± 18.97	55.83 ± 17.84	< 0.001
**Pharmaceutical delivery**, *N (%)*				**< 0.001**
< 18000	448,684 (67.1)	263,484 (65.4)	185,200 (60.2)	
18000–100000	188209 (28,1)	77950 (21.6)	110259 (35.8)	
> 100000	2907 (0.4)	1093 (0.3)	1814 (0.6)	
Others	39174 (4.4)	18624 (5.2)	10550 (3.4)	
**Basic health area** N (%)				**< 0.001**
Urban	356107 (53.2)	194275 (53.8)	161832 (52.6)	
Semiurban/Rural	312864 (46.8)	166875 (46.2)	145989 (47.4)	
**Chronic illness (Yes %)**, N (%)				
Arrhythmias	40104 (6)	19666 (5.4)	20438 (6.6)	**< 0.001**
Heart failure	14256 (2.1)	8152 (2.3)	6104 (2.0)	**< 0.001**
Ischaemic heart disease	28753 (4.3)	9260 (2.6)	19493 (6.3)	**< 0.001**
Hypertension	234823 (35.1)	122275 (33.9)	112548 (36.6)	**< 0.001**
Dyslipidaemia	281528 (42.1)	144144 (39.9)	137384 (44.6)	**< 0.001**
Obesity	85809 (12.8)	49649 (13.7)	36161 (11.7)	**< 0.001**
Vein/artery disease	17106 (2.6)	7248 (2)	9858 (3.2)	**< 0.001**
Cerebrovascular disease	27703 (4.1)	13861 (3.8)	13842 (4.5)	**< 0.001**
Diabetes	82377 (12.3)	36341 (10.1)	46036 (15.0)	**< 0.001**
Chronic bronchitis	7109 (1.1)	3606 (1)	3503 (1.1)	**< 0.001**
COPD	22957 (1.1)	6818 (1.9)	16139 (5.2)	**< 0.001**
Asthma	58949 (8.8)	35159 (9.7)	23790 (7.7)	**< 0.001**
Chronic kidney disease	34466 (5.2)	19041 (5,3)	15425 (5.0)	**< 0.001**
Smoking	140076 (20.9)	63082 (17.5)	7699 (25.0)	**< 0.001**
Alcoholism	10462 (1.6)	1658 (0.5)	8804 (2.9)	**< 0.001**
Insomnia	86067 (12.9)	51251 (14,2)	34816 (11.3)	**< 0.001**
Anxiety and depression	231203 (34.6)	156935 (43,5)	74268 (24.1)	**< 0.001**
Autolytic attempt	2428 (0.4)	1460 (0.4)	968 (0.3)	**< 0.001**
Anaemia	87604 (13.1)	64178 (17.8)	23426 (7.6)	**< 0.001**
Neoplasia	163426 (24.4)	97707 (27.1)	65719 (21.3)	**< 0.001**
Dementia	13558 (2.0)	9412 (2.6)	4146 (1.3)	**< 0.001**
Hearing loss	47572 (7.1)	14586 (6.8)	22986 (7.5)	**< 0.001**
Cataracts	58764 (8.8)	34945 (9.7)	23819 (7.7)	**< 0.001**
Glaucoma	38479 (5.8)	22217 (6.2)	16262 (5.3)	**< 0.001**
**Number of chronic illnesses.** Mean ± SD	3.12 ± 2.10	3.19 ± 2.17	3.00 ± 1.96	**< 0.001**
1, N *(%)*	139204 (20.8)	60560 (16.8)	78644 (25.5)	**< 0.001**
2–4, N *(%)*	363609 (54.4)	195249 (54.1)	168360 (54.7)	
5–9, N *(%)*	158884 (23.6)	100195 (27.7)	58689 (19.1)	
10 or more, N *(%)*	7277 (1.2)	5147 (1.4)	2130 (0.7)	

SD Standard deviation; COPD Chronic Obstructive Pulmonary Disease. Bold values indicate that the results are statistically significant.

**Table 2:** New diagnoses of other chronic comorbidities in the cohort during the six months before the lockdown, the first six months and from six to twelve months after the lockdown

New diagnosis of chronic comorbidities (Yes %), *N (%)*	6 months before lockdown (n = 662,754)	0–6 months after lockdown (n = 654,954)	6–12 months after lockdown (n = 649,702)	Cochran’s Q test, P
Arrhythmias	2965 (0.4)	1864 (0.3)	667 (0.1)	**< 0.001**
Heart failure	1184 (0.2)	924 (0.1)	316 (0.03)	**< 0.001**
Ischaemic heart disease	1914 (0.3)	1483 (0.2)	530 (0.1)	**< 0.001**
Hypertension	3382 (0.5)	2011 (0.3)	956 (0.1)	**< 0.001**
Dyslipidaemia	4707 (0.7)	2741 (0.4)	1105 (0.2)	**< 0.001**
Obesity	1269 (0.2)	512 (0.1)	204 (0.05)	**< 0.001**
Vein/artery disease	683 (0.1)	391 (0.05)	167 (0.02)	**< 0.001**
Cerebrovascular disease	1588 (0.2)	1349 (0.2)	436 (0.06)	**< 0.001**
Diabetes	1709 (0.3)	1181 (0.2)	560 (0.1)	**< 0.001**
Chronic bronchitis	273 (0.04)	132 (0.02)	43 (0.006)	**< 0.001**
COPD	723 (0.1)	230 (0.03)	82 (0.01)	**< 0.001**
Asthma	653 (0.09)	318 (0.04)	71 (0.01)	**< 0.001**
Chronic kidney disease	2469 (0.4)	2022 (0.3)	646 (0.1)	**< 0.001**
Smoking	3208 (0.47)	914 (0.13)	285 (0.04)	**< 0.001**
Alcoholism	316 (0.04)	238 (0.03)	63 (0.009)	**< 0.001**
Insomnia	4539 (0.7)	3427 (0.5)	1107 (0.2)	**< 0.001**
Anxiety and depression	10,549 (1.6)	9287 (1.4)	1807 (0.3)	**< 0.001**
Autolytic attempt	170 (0.02)	149 (0.02)	72 (0.01)	**< 0.001**
Anaemia	4458 (0.7)	3309 (0.5)	1083 (0.2)	**< 0.001**
Neoplasia	9817 (1.46)	6545 (1.0)	2048 (0.3)	**< 0.001**
Dementia	1328 (0.2)	1075 (0.2)	356 (0.05)	**< 0.001**
Hearing loss	2238 (0.3)	1177 (0.2)	393 (0.05)	**< 0.001**
Cataracts	3691 (0.6)	2360 (0.4)	733 (0.1)	**< 0.001**
Glaucoma	1042 (0.15)	496 (0.07)	224 (0.03)	**< 0.001**

Bold values indicate that the results are statistically significant.

Regarding new diagnoses of chronic comorbidities, as shown in Table 2, there are fewer new diagnoses during the first six months and from six to twelve months after the lockdown compared to six months before the pandemic. A decrease in new diagnoses of chronic comorbidities was observed in the last period analysed (from six to twelve months).

When comparing gender, a decrease was observed in the complete sample of diagnoses as the pandemic progressed. New diagnoses of chronic illnesses, which are more frequent during the two first periods, were COPD in men and depression in women. However, this difference tends to decrease during the time frame from six to twelve months following the start of the pandemic. Although new diagnoses decrease, some diseases do not do so as abruptly as others. (Supplementary 2)

Regarding the mortality rate (excludes COVID-19), as shown in Table 3, it is observed. The mortality rate per 1,000 individuals was higher in men with chronic diseases than in women (p-value: <0.001), except during the 6-month post-lockdown period, which was higher in women. For both, the mortality rate decreased as the pandemic progressed (p-value: <0.001).

**Table 3:** Comparison by sex of the mortality rate during six months before the lockdown, the first six months and from six to twelve months after the lockdown

	6 months before lockdown (n = 662,754)				0–6 months after lockdown (N = 654,954)				6–12 months after lockdown (N = 649,702)			
	*Total*	*Men*	*Women*	*p-value*	*Total*	*Men*	*Women*	*p-value*	*Total*	*Men*	*Women*	*p-value*
Died people, *N*	6,220	3,141	3,079	**< 0.001**	5,719	2,773	2,946	**< 0.001**	5,252	2,637	2,615	**< 0.001**
Mortality rate % per 1,000 individuals	5.49	5.55	5.33	**< 0.001**	5.04	4.99	5.09	**< 0.001**	4.62	4.74	4.51	**< 0.001**

No. Number: Bold values indicate that the results are statistically significant

**Table 4:** Comparison by sex of the difference of active ingredients and cost during six months before the lockdown, the first six months and from six to twelve months after the lockdown

	6 months before lockdown (n = 662,754)				0–6 months after lockdown (N = 654,954)				6–12 months after lockdown (N = 649,702)			
	*Total*	*Men*	*Women*	*p-value*	*Total*	*Men*	*Women*	*p-value*	*Total*	*Men*	*Women*	*p-value*
People with prescribed medications, *N*	*380,774*	163,080	217,694	**< 0.001**	*370,876*	157,973	212,903	**< 0.001**	*364,300*	154,920	209,380	**< 0.001**
No. of active ingredients, *Mean ± SD*	2.64 *±* 1.77	2.56 ± 1.80	2.52 ± 1.72	**< 0.001**	2.46 *±* 1.61	2.37 ± 1.61	2.39 ± 1.57	**< 0.001**	2.17 *±* 1.56	2.35 ± 1.31	2.38 ± 1.63	**< 0.001**
Cost of active ingredients, *Mean ± SD*	81.58 *±* 124.25	91.70 ± 139.06	64.60 ± 102.88	**< 0.001**	81.33 *±* 123.33	91.67 ± 138.98	64.85 ± 103.11	**< 0.001**	67.46 *±* 102.61	76.72 ± 115.76	54.46 ± 86.12	**< 0.001**

No. Number; SD Standard deviation; Bold values indicate the results are statistically significant.

About the medications dispensed by the pharmacy, as shown in Table 4, there is a slight decrease in both the number of active ingredients and cost as the pandemic progresses. Regarding gender, although women have a more significant number of diagnoses of chronic diseases, the number of medications dispensed is lower.

The use of the study population of health resources can be seen in Table 5. Between the six months before and after the lockdown, the number of visits to healthcare professionals decreased, except for the number of nurse visits at home and GP visits at the health centre or via telephone. Between six and twelve months following the lockdown, there was a considerable reduction in the use of health services, except for the number of visits to A&E, which remained stable. The number of hospital admissions and the number of days at the ICU increased notably. When comparing gender, as shown in Table 6, the frequency and percentage of women who used health services during the three-time frame were higher than for men, except for hospital and ICU admissions, which were higher for men.

**Table 5:** Comparations by sex of the number of health services during six months before the pandemic’s beginning, six months after the lockdown, and six to twelve months after the lockdown

	Differences 6 months before − 6 months after lockdown (N = 654,954)				Difference 0–6 and 6–12 months after lockdown (N = 649,702)			
	Six months before *Mean ± SD*	0–6 months after lockdown *Mean ± SD*	*N*	*p-value*	0–6 months after lockdown *Mean ± SD*	6–12 months after lockdown *Mean ± SD*	*N*	*p-value*
**No. of visits Nursing**,								
At health centre or by telephone (ordinary care)	4.01 ± 4.68	3.51 ± 4.20	215247	**< 0.001**	3.84 ± 4.46	3.55 ± 3.92	182840	**< 0.001**
At home (ordinary care)	6.66 ± 9.86	6.17 ± 9.43	15422	**< 0.001**	4.83 ± 8.07	3.81 ± 7.69	24735	**< 0.001**
At health centre (continuous care)	2.01 ± 2.76	1.76 ± 2.43	14046	**< 0.001**	2.04 ± 2.97	1.77 ± 2.43	12442	**< 0.001**
At home (continuous care)	2.21 ± 3.17	2.29 ± 4.92	1981	**< 0.001**	2.30 ± 5.23	1.69 ± 3.7	2563	**< 0.001**
**No. of visits General practitioner**								
At health centre or by telephone (ordinary care)	4.81 ± 4.33	4.90 ± 4.73	420597	**< 0.001**	5.03 ± 4.81	4.56 ± 4.1	395165	**< 0.001**
At home (ordinary care)	3.26 ± 3.43	2.56 ± 2.79	3892	**< 0.001**	1.84 ± 1.99	0.98 ± 2.11	19978	**< 0.001**
At health centre (continuous care)	1.81 ± 1.70	1.76 ± 1.83	34347	**< 0.001**	2.05 ± 2.29	1.73 ± 1.6	29544	**< 0.001**
At home (continuous care)	1.72 ± 1.42	1.35 ± 0.63	2199	**< 0.001**	1.43 ± 0.74	0.86 ± 1.25	3211	**< 0.001**
**No. of visits to other professionals**								
Physiotherapist	6.32 ± 6.66	4.31 ± 4.91	2358	**< 0.001**	4.54 ± 5.05	3.40 ± 4.77	3444	**< 0.001**
Midwife	3.15 ± 3.53	3.46 ± 3.47	4523	**< 0.001**	3.08 ± 3.11	2.96 ± 3.38	5540	**0.023**
Dentist	2.04 ± 1.59	2.18 ± 1.57	1550	**< 0.001**	2.14 ± 1.57	1.26 ± 1.52	2001	**0.007**
Social worker	2.60 ± 2.67	3.04 ± 3.45	1616	**< 0.001**	2.98 ± 3.53	2.28 ± 3.14	2127	**< 0.001**
**No. of visits to Specialised care**								
First consultation	1.43 ± 0.81	1.49 ± 0.88	15195	**< 0.001**	1.46 ± 0.85	0.55 ± 0.84	37795	**< 0.001**
Successive consultations	2.55 ± 2.18	2.49 ± 2.31	126843	**< 0.001**	2.58 ± 2.38	2.25 ± 2.14	118422	**< 0.001**
**No. of visits to the emergency department**	1.73 ± 1.41	1.64 ± 1.31	23950	**< 0.001**	1.67 ± 1.36	1.66 ± 1.28	19970	0.587
**Hospital**								
No. of admissions	1.25 ± 0.65	0.23 ± 0.64	27239	**< 0.001**	0.27 ± 0.69	1.27 ± 0.65	27529	**< 0.001**
No. of days stay	22.62 ± 158.71	21.69 ± 156.14	4339	**0.034**	18.03 ± 80.77	22.35 ± 124.33	5292	**0.001**
**ICU**								
No. of admissions	1 ± 0	1.06 ± 0.25	15	0.317	1.04 ± 0.20	1 ± 0	24	0.317
No. of days stay	65.26 ± 64.17	57.20 ± 64.08	15	0.575	31.50 ± 22.72	41.91 ± 30.30	24	**0.014**

No. Number; SD Standard deviation; ICU Intensive care unit; Bold values indicate statistically significant results.

**Table 6:** Frequency and percentage of women and men that utilised health services during six months before the beginning of the pandemic, six months after the lockdown, and six to twelve months after the lockdown

		6 months before the lockdown (n = 662,754),			0–6 months after lockdown (N = 654,954)			6–12 months after lockdown (N = 649,702)		
		Men, *N (%)* N = 304,744	Women, *N (%)* N = 358,010	*P-value*	Men, *N (%)* N = 300,984	Women, *N (%)* N = 353,970	*P-value*	Men, *N (%)* N = 298,347	Women, *N (%)* N = 351,355	*P-value*
**Nursing visit** (Yes %)										
	At health centre or by telephone (ordinary care)	139,132 (45.2)	177,055 (49.0)	**< 0.001**	137,074 (44.5)	175,134 (48.5)	**< 0.001**	118,908 (38.6)	154,526 (42.8)	**< 0.001**
	At home (ordinary care)	11,155 (3.6)	19,895 (5.5)	**< 0.001**	12,059 (3.9)	21,257 (5.9)	**< 0.001**	9549 (3.1)	16,764 (4.6)	**< 0.001**
	At health centre (continuous care)	30,796 (10.0)	39,699 (11.0)	**< 0.001**	23,436 (7.6)	27,795 (7.7)	0.205	21,849 (7.1)	26,623 (7.4)	**< 0.001**
	At home (continuous care)	4871 (1.6)	6833 (1.9)	**< 0.001**	3803 (1.2)	5125 (1.4)	**< 0.001**	3479 (1.1)	4661 (1.3)	**< 0.001**
**General practitioner** (Yes %)										
	At health centre or by telephone (ordinary care)	224,507 (72.9)	294,224 (81.5)	**< 0.001**	210,049 (68.2)	278,392 (77.1)	**< 0.001**	201,956 (65.6)	271,690 (75.2)	**< 0.001**
	At home (ordinary care)	11,692 (3.8)	20,487 (5.7)	**< 0.001**	8696 (2.8)	15,161 (4.2)	**< 0.001**	7907 (2.6)	13,619 (3.8)	**< 0.001**
	At health centre (continuous care)	52,307 (17.0)	72,857 (20.2)	**< 0.001**	41,610 (13.5)	56,113 (15.5)	**< 0.001**	35,780 (11.6)	49,178 (13.6)	**< 0.001**
	At home (continuous care)	5669 (1.8)	8402 (2.3)	**< 0.001**	4091 (1.3)	5678 (1.6)	**< 0.001**	3704 (1.2)	5248 (1.5)	**< 0.001**
**Visit to other professionals** (Yes %)										
	Physiotherapist	4468 (1.5)	9892 (2.7)	**< 0.001**	3349 (1.1)	7313 (2.0)	**< 0.001**	3816 (1.2)	8264 (2.3)	**< 0.001**
	Dentist	5061 (1.6)	6329 (1.8)	**0.001**	3405 (1.1)	4049 (1.1)	0.561	3182 (1.0)	4245 (1.2)	**< 0.001**
	Social worker	3224 (1.0)	5621 (1.6)	**< 0.001**	2550 (0.8)	4443 (1.2)	**< 0.001**	2905 (0.9)	5016 (1.4)	**< 0.001**
**Specialised care** (Yes %)										
	First consultation	30,339 (9.9)	39,903 (11.0)	**< 0.001**	22,923 (7.4)	31,795 (8.8)	**< 0.001**	25,476 (8.3)	34,685 (9.6)	**< 0.001**
	Successive consultations	94,016 (30.5)	126,321 (35.0)	**< 0.001**	79,674 (25.9)	105,922 (29.3)	**< 0.001**	77,998 (25.3)	105,603 (29.2)	**< 0.001**
**Visit to the emergency department** (Yes %)		45,077 (14.6)	59,322 (16.4)	**< 0.001**	33,174 (10.8)	43,314 (12.0)	**< 0.001**	32,627 (10.6)	43,598 (12.1)	**< 0.001**
**Hospital admissions** (Yes %)		13,167 (4.3)	14,072 (3.9)	**< 0.001**	13,223 (4.3)	14,232 (3.9)	**< 0.001**	13,232 (4.3)	14,297 (4.0)	**< 0.001**
**ICU admissions** (Yes %)		553 (0.2)	410 (0.1)	**< 0.001**	400 (0.12)	251 (0.07)	**< 0.001**	310 (0.10)	182 (0.05)	**< 0.001**

ICU Intensive care unit. Bold values indicate that the results are statistically significant.

## Discussion

This study analysed the new comorbidities, mortality, medication for chronic pathologies use and health resources of patients with chronic diseases in an autonomous community in Southern Europe (Aragon, Spain) who did not contract COVID-19. The decrease in the use of PHC health services observed in our study as the pandemic progresses is a universal phenomenon confirmed in different surveillance systems in other places (Europe, Australia, and Canada) [29]. Various world governments implemented restrictive measures to avoid the collapse of health services, focusing on hospital health care and the consequent closure of health centres to the population [30] With the closure of health centres during lockdown for face-to-face activities, standard follow-up visits for chronic pathologies or new diagnoses of these (analytics, examinations), a decrease of up to 50% in new diagnoses for some chronic pathologies such as hypertension and COPD concerning data from previous years, which coincides with the findings of this study [15, 31]. The only activity maintained in some cases was urgent care (acute care, treatment, control of anticoagulants, etc.). Thus, there was no exacerbated decrease in new urgent diagnoses such as strokes, heart attacks, etc. [15, 31]. However, a reduction in care and new stroke diagnoses have been observed in different countries, similar to what was obtained in our results [32, 33]. In addition, the reduction in the use of emergency services may be related to the fear of patients of possible contamination related to SARS-CoV-2 if they are transported to the hospital [34]. However, the decrease in the control of patients with chronic conditions during confinement led to a lack of follow-up sessions and the prevention of the disease worsening, with a consequent increase in symptoms and the aggravation of the chronic pathologies of the patients with a severe evolution, for which a referral and hospital admission was required following the lockdown [15, 31, 35].

A recent study by Lear-Claveras et al. carried out in a population with hypertension, showed a decrease in the use of services, worsening symptoms, and clinical parameters [36]. A possible longer diagnostic and therapeutic delay of other severe and emerging pathologies during the pandemic in women, or worse control and follow-up of underlying chronic diseases, may be associated with a decrease in mortality rates from other causes during this period. The data verified by the Spanish National Institute of Statistics from 2019 to 2020 are consistent with the trend observed in our study regarding gender and sex, particularly in the causes of mortality not due to COVID-19 [37]. The rates of mortality decrease as the pandemic continues (both in women and men) but are lower in women than in men, except in the time frame from zero to six months post-lockdown. However, these findings do not correlate with previous studies reporting a higher mortality rate [not caused by COVID-19] in women during the first wave. However, they report a higher rate in women during the post-lockdown period [38]. The UK national reports confirm this significant decrease in non-COVID-19-related mortality rates. The most considerable reductions in percentage terms are deaths from influenza/pneumonia (48%) and chronic lung disease (25%). These reductions are due in large part to the lower prevalence of infectious diseases as a result of social distancing and mask-wearing [39].

There is evidence of lockdown’s impact on clinical variables in specific chronic pathologies, such as diabetes or arterial hypertension. Still, the set of chronic diseases was not considered from a gender perspective [40, 41]. In a recent report from the Spanish Ministry of Equality, as shown by our results, they reveal that women go to health services more frequently than men. This may be due to greater longevity and a high rate of comorbidities in women. In addition, women are not only frequenters of health services for their health but also because of the increased presence of women as caregivers of the elderly or sick [42].

Currently, the number of studies conducted to observe the impact of the COVID-19 contagion in patients with persistent comorbidities is rising [43–45]. In contrast, our large-scale longitudinal study was conducted on a demographic with chronic diseases whom COVID-19 had not infected.

Our study has some limitations. In the first instance, the large sample size in this study means that minor variations are statistically significant, no matter how insignificant. Therefore, it is necessary to be careful when interpreting the results and observing the data obtained within the logic.

Secondly, our study carried out a longitudinal follow-up of up to one year. Still, it is necessary to conduct more long-term studies since, due to the saturation of PHC consultations or the fear of contagion from chronically ill patients, these new diagnoses and consequences present a posteriori.

Thirdly, we do not know whether the electronic health record of the patients included the date of the diagnosis of their chronic pathology, and, therefore, we do not know its course and severity nor the consequent need for the number of visits required for its control. Also, it is essential to consider that the data is collected from the electronic record of primary health care, so it may be that the records made from the emergency room or hospital have not been compiled with the same diagnostic labelling system as from PHC.

Lastly, this study did not include self-reported data on lifestyle habits and self-perceived control of symptoms maintained by the study population during the months of lockdown.

Before the pandemic, public health initiatives concentrated on streamlining the healthcare system’s coordination and offering recommendations for managing chronic illnesses and lifestyle variables. However, given the unprecedented situation brought on by the COVID-19 pandemic, structural changes to the health systems—including the one in Spain—may be necessary to give priority to actions aimed at bettering the management of chronic care, addressing the fundamental needs of patients with chronic illnesses, and reducing the potentially disastrous effects of the COVID-19 outbreak on particularly vulnerable individuals. The PHC serves as the population’s nearest community health resource and the entrance to the healthcare system. Therefore, epidemiological analysis using real-world data (RWD), such as data from PHC records, provides results related to decision-making from a healthcare perspective.

## Conclusion

This study showed an underdiagnosis of new chronic comorbidities and a decrease in mortality rates unrelated to COVID-19 due to the closure of health centres in Aragón (Spain) during the lockdown. Sex influences this tendency, which is more exacerbated in women. In addition, a decrease in the prescription of medications and the use of PHC services was evidenced as the pandemic progressed. The underdiagnosis and the reduction in control of patients with chronic diseases during lockdown can worsen the condition, with the consequent increase in symptoms and the worsening of chronic pathologies of patients with a severe evolution. Structural changes are needed in healthcare systems to prioritise actions aimed at improving chronic care management, addressing the fundamental needs of chronically ill patients, and decreasing the potentially disastrous effects of the COVID-19 outbreak on particularly vulnerable people.

### Electronic supplementary material

Below is the link to the electronic supplementary material.

Supplementary Material 1

## Data Availability

The datasets and materials used and/or analysed during the current study are available from the corresponding author upon reasonable request.

## Abbreviations

AHA: American Heart Association
A&E: Accident and emergency department
COPD: Chronic Obstructive Pulmonary Disease
GP: General practitioner
ICPC-2: International Classification of Primary Care
ICU: Intensive care unit
No.: Number
PHC: Primary health care
RWD: Real-world data
SD: Standard deviation
WHO: World Health Organization
