# Supplementary material for: Use of health services and medication use, new comorbidities, and mortality in patients with chronic diseases who did not contract COVID-19 during the first year of the pandemic: a retrospective study and comparison by sex
Source: BMC Health Serv Res. 2023 Dec 6;23:1364. doi: 10.1186/s12913-023-10158-7 (PMC10698932; doi:10.1186/s12913-023-10158-7)
Supplement: Supplementary file 1 — Supplementary Material 1 [file 12913_2023_10158_MOESM1_ESM.docx]

**Supplementary 1 Population pyramid of sample study**

1. Six months before the start of lockdown (from 14th September 2019 to 15th March 2020)(n = 662,754)

B) Six months following the end of strict lockdown (from 3rd May to 4th November 2020) (n = 654,954)

C ) Six to 12 months after the lockdown (from 5th November 2020 to 6th May 2021) (n = 649,702).

**Supplementary 2 Population pyramid of population of the same age range that has suffered COVID-19**

A ) Six months following the end of strict lockdown (from 3rd May to 4th November 2020)

B ) Six to 12 months after the lockdown (from 5th November 2020 to 6th May 2021)

**Supplementary 3: New diagnoses of chronic comorbidities 6 months before the beginning of the pandemic, 6 months after the lockdown, and from 6 to 12 months after the lockdown, comparing by sex.**

| New diagnoses of chronic comorbities (Yes %) | | Women N(%) | Men N(%) | P-value |  |
| --- | --- | --- | --- | --- | --- |
| **Arrhythmias** | |  |  |  |  |
|  | 6 months before pandemic  0 to 6 months after lockdown  6 to 12 months after lockdown | 1458 (0.4)  929 (0.3)  334 (0.1) | 1507 (0.5)  965 (0.3)  343 (0.1) | **<0.001**.  **<0.001**  **<0.001** |  |
| **Heart** **failure** | |  |  |  |  |
|  | 6 months before pandemic  0 to 6 months after lockdown  6 to 12 months after lockdown | 655 (0.2)  511 (0.1)  160 (0.04) | 529 (0.2)  413 (0.1)  156 (0.09) | .356  .421  .232 |  |
| **Ischaemic heart disease** | |  |  |  |  |
|  | 6 months before pandemic  0 to 6 months after lockdown  6 to 12 months after lockdown | 918 (0.3)  690 (0.2)  225 (0.1) | 1022 (0.3)  793 (0.2)  305 (0.1) | **<0.001**.  **<0.001**  **<0.001** |  |
| **Hypertension** | |  |  |  |  |
|  | 6 months before pandemic  0 to 6 months after lockdown  6 to 12 months after lockdown | 1688 (0.5)  1073 (0.3)  505 (0.1) | 1694 (0.6)  938 (0.3)  451 (0.1) | **.001**  .571  .471 |  |
| **Dyslipidaemia** | |  |  |  |  |
|  | 6 months before pandemic  0 to 6 months after lockdown  6 to 12 months after lockdown | 2465 (0.7)  1770 (0.5)  690 (0.2) | 1942 (0.6)  971 (0.3)  415 (0.1) | **.009**  **<0.001**  **<0.001** |  |
| **Obesity** | |  |  |  |  |
|  | 6 months before pandemic  0 to 6 months after lockdown  6 to 12 months after lockdown | 712 (0.2)  300 (0.1)  111 (0.03) | 557 (0.2)  212 (0.1)  93 (0.04) | .129  .036  .903 |  |
| **Overweight** | | | | | |
|  | 6 months before pandemic  0 to 6 months after lockdown  6 to 12 months after lockdown | 197 (0.1)  67 (0.018)  23 (0.006) | 85 (0.02)  33 (0.01)  8 (0.002) | **<0.001**  .**009**  **.024** |  |
| **Vein/artery disease** | | | | | |
|  | 6 months before pandemic  0 to 6 months after lockdown  6 to 12 months after lockdown | 281 (0.1)  133 (0.03)  68 (0.01) | 402 (0.13)  258 (0.08)  99 (0.01) | **<0.001**.  **<0.001**  **<0.001** |  |
| **Cerebrovascular** **disease** | | | | | |
|  | 6 months before pandemic  0 to 6 months after lockdown  6 to 12 months after lockdown | 890 (0.2)  712 (0.2)  237 (0.1) | 698 (0.2)  637 (0.2)  199 (0.1) | **.009**  .374  .876 |  |
| **Diabetes** | | | | | |
|  | 6 months before pandemic  0 to 6 months after lockdown  6 to 12 months after lockdown | 751 (0.2)  529 (0.14)  253 (0.07) | 958 (0.3)  652 (0.2)  307 (0.1) | **<0.001**.  **<0.001**  **<0.001** |  |
| **Chronic bronchitis** | | | | | |
|  | 6 months before pandemic  0 to 6 months after lockdown  6 to 12 months after lockdown | 142 (0.03)  82 (0.02)  22 (0.006) | 134 (0.04)  50 (0.016)  21 (0.006) | .398  .061  .710 |  |
| **COPD** | | | | | |
|  | 6 months before pandemic  0 to 6 months after lockdown  6 to 12 months after lockdown | 261 (0.1)  96 (0.02)  29 (0.008) | 462 (0.2)  134 (0.04)  53 (0.01) | **<0.001**  **<0.001**  **.001** |  |
| **Asthma** | | | | | |
|  | 6 months before pandemic  0 to 6 months after lockdown  6 to 12 months after lockdown | 469 (0.12)  224 (0.06)  53 (0.01) | 184 (0.1)  94 (0.03)  18 (0.005) | **<0.001**  **<0.001**  **<0.001** |  |
| **Chronic kidney disease** | | | | | |
|  | 6 months before pandemic  0 to 6 months after lockdown  6 to 12 months after lockdown | 1366 (0.4)  1174 (0.3)  351 (0.1) | 1103 (0.4)  848 (0.3)  295 (0.1) | .181  **<0.001**  .859 |  |
| **Hypothyroidism** | | | | | |
|  | 6 months before pandemic  0 to 6 months after lockdown  6 to 12 months after lockdown | 1356 (0.4)  966 (0.3)  358 (0.1) | 528 (0.2)  392 (0.1)  146 (0.04) | **<0.001**.  **<0.001**  **<0.001** |  |

| New diagnoses of chronic comorbities (Yes %) | | | Women N(%) | Men N(%) | P-value |  |
| --- | --- | --- | --- | --- | --- | --- |
| **Hyperthyroidism** | | |  |  |  |  |
|  | | 6 months before pandemic  0 to 6 months after lockdown  6 to 12 months after lockdown | 838 (0.23)  560 (0.15)  191 (0.05) | 259 (0.08)  183 (0.05)  53 (0.01) | **<0.001**.  **<0.001**  **<0.001** |  |
| **Smoking** | | |  |  |  |  |
|  | 6 months before pandemic  0 to 6 months after lockdown  6 to 12 months after lockdown | | 1582 (0.4)  444 (0.1)  142 (0.03) | 1626 (0.5)  471 (0.2)  143 (0.04) | **<0.001**  .001  .159 |  |
| **Alcoholism** | | |  |  |  |  |
|  | 6 months before pandemic  0 to 6 months after lockdown  6 to 12 months after lockdown | | 84 (0.02)  71 (0.019)  21 (0.005) | 232 (0.07)  167 (0.05)  42 (0.001) | **<0.001**  **.001**  **<0.001** |  |
| **Insomnia** | | |  |  |  |  |
|  | 6 months before pandemic  0 to 6 months after lockdown  6 to 12 months after lockdown | | 2430 (0.7)  1903 (0.5)  644 (0.2) | 2109 (0.7)  1524 (0.5)  463 (0.2) | .543  .069  **.005** |  |
| **Anxiety and depression** | | |  |  |  |  |
|  | 6 months before pandemic  0 to 6 months after lockdown  6 to 12 months after lockdown | | 7267 (2)  6489 (1.8)  1246 (0.3) | 3282 (1.1)  2798 (0.9)  561 (0.2) | **<0.001**.  **<0.001**  **<0.001** |  |
| **Autolytic attempt** | | |  |  |  |  |
|  | 6 months before pandemic  0 to 6 months after lockdown  6 to 12 months after lockdown | | 105 (0.02)  98 (0.02)  25 (0.006) | 65 (0.02)  61 (0.01)  17 (0.005) | **.042**  .053  .471 |  |
| **Anaemia** | | |  |  |  |  |
|  | 6 months before pandemic  0 to 6 months after lockdown  6 to 12 months after lockdown | | 2852 (0.8)  2255 (0.6)  668 (0.2) | 1606 (0.5)  1354 (0.4)  415 (0.1) | **<0.001**.  **<0.001**  **<0.001** |  |
| **Neoplasia** | | |  |  |  |  |
|  | 6 months before pandemic  0 to 6 months after lockdown  6 to 12 months after lockdown | | 5408 (1.49)  3633 (1)  1146 (0.3) | 4409 (1,43)  2,962 (1)  902 (0.3) | **.031**  .071  .073 |  |
| **Dementia** | | |  |  |  |  |
|  | 6 months before pandemic  0 to 6 months after lockdown  6 to 12 months after lockdown | | 841 (0.23)  689 (0.19)  219 (0.1) | 487 (0.2)  386 (0.1)  137 (0.04) | **<0.001**  **<0.001**  .**004** |  |
| **Hearing loss** | | | | | | |
|  | 6 months before pandemic  0 to 6 months after lockdown  6 to 12 months after lockdown | | 1231 (0.3)  640 (0.2)  230 (0.1) | 1007 (0.3)  537 (0.2)  163 (0.1) | .333  .788  .071 |  |
| **Cataracts** | | | | | | |
|  | 6 months before pandemic  0 to 6 months after lockdown  6 to 12 months after lockdown | | 2079 (0.6)  1343 (0.4)  390 (0.1) | 1612 (0.5)  1017 (0.3)  343 (0.1) | **.004**  **.004**  .672 |  |
| **Glaucoma** | | | | | | |
|  | 6 months before pandemic  0 to 6 months after lockdown  6 to 12 months after lockdown | | 605 (0.2)  234 (0.1)  123 (0.03) | 437 (0.14)  222 (0.07)  101 (0.03) | **.008**  .574  .781 |  |
| **Osteoarthritis** | | | | | | |
|  | 6 months before pandemic  0 to 6 months after lockdown  6 to 12 months after lockdown | | 927 (0.3)  591 (0.2)  479 (0.1) | 502 (0.2)  287 (0.1)  209 (0.1) | **<0.001**.  **<0.001**  **<0.001** |  |
| **Osteoporosis** | | | | | | |
|  | 6 months before pandemic  0 to 6 months after lockdown  6 to 12 months after lockdown | | 630 (0.2)  359 (0.1)  137 (0.03) | 69 (0.02)  45 (0.01)  23 (0.007) | **<0.001**.  **<0.001**  **<0.001** |  |
| **Dorsopathy** | | | | | | |
|  | 6 months before pandemic  0 to 6 months after lockdown  6 to 12 months after lockdown | | 3812 (1.1)  2691 (0.7)  914 (0.3) | 2224 (0.7)  1616 (0.5)  510 (0.2) | **<0.001**.  **<0.001**  **<0.001** |  |
